# Insight in the Recent Application of Polyphenols From Biomass

**DOI:** 10.3389/fbioe.2021.753898

**Published:** 2021-09-13

**Authors:** Bowen Yan, Zhefan Stephen Chen, Yingying Hu, Qiang Yong

**Affiliations:** ^1^ Co-Innovation Center for Efficient Processing and Utilization of Forest Products, College of Chemical Engineering, Nanjing Forestry University, Nanjing, China; ^2^ Nexus of Rare Neurodegenerative Diseases, School of Life Sciences, Faculty of Science, The Chinese University of Hong Kong, Hong Kong, SAR China

**Keywords:** biomass, polyphenols, biological application, bioactivities, health functions

## Abstract

Biomass polyphenols are bio-active macromolecules with distinct chemical structures in a variety of biomass. In recent years, the study of biomass polyphenols and their application in food and medicine fields has become a research hotspot, which predominantly focuses on the preparation, purification, structural identifications, and measurements of biological activities. Many studies describe methodologies for extraction and application of polyphenols, but comprehensive work to review its physiological activities like drugs and health products are lacking. This paper comprehensively unlocks the bioactivities of antioxidant, antibacterial, antitumor, anticancer, neuroprotection, control of blood sugar, regulation of blood fat, and promotion of gastrointestinal health functions of polyphenols from different biomass sources. This review will serve as an illuminating resource for the global scientific community, especially for those who are actively working to promote the advances of the polyphenols research field.

## Introduction

The growth and development of biomass are sustained by the regulation of metabolism and resistance against different biological stresses. The endogenous metabolites of biomass are composed of primary and secondary metabolites. In the different biomass, the major constituents are cellulose, hemicellulose, lignin, which can be applied to prepare various value-added products and bio-materials (Sun et al., 2019; Zhou et al., 2019; Chen et al., 2020; Geng et al., 2020; Chen et al., 2021; Liu et al., 2021; Luo et al., 2021). Apart from these constituents, there is existed various minor constituents, such as flavonoids and polyphenols (Si et al., 2009; Gironi and Piemonte, 2011; Quideau et al., 2011; Goodman, 2020). Biomass polyphenols are one type of secondary metabolites synthesized primarily through the shikimic acid and phenylpropane pathways (Hidalgo-Liberona et al., 2020; Wang et al., 2020). Biomass polyphenols are widely found in plant skins, roots, leaves, and fruits, with an abundance of as much as 20% by weight (Quideau et al., 2011).

There has been a long history of utilization of biomass polyphenols, which have been used in tanning and as medicines starting from ancient times (Quideau et al., 2011). The natural feelings and intrinsic properties of biomass polyphenols make them remarkable amongst plant-derived products. In recent years, biomass polyphenols have attracted much more attention in green/sustainable scientific fields due to their broad distribution, natural abundance, diverse chemical structures, and biological functions. A range of studies has demonstrated that biomass polyphenols comprise multiple phenolic hydroxyl groups, which have been reported to elicit prominent physiological functions such as free radical scavenging and radical sequestration activities (Gironi and Piemonte, 2011; Dong et al., 2020; Pei et al., 2020). These functionalities thus highlight biomass polyphenols as effective antioxidants. In addition to executing antioxidant activities, biomass polyphenols dramatically inhibit the growth of different strains of bacteria, fungi, and viruses while not affecting the growth and development of beneficial microorganisms under weak acidic and neutral environments. This indicates the potential applications of biomass polyphenols as bacteriostatic and anti-tumor agents. Moreover, biomass polyphenols also effectively protect against cardiovascular diseases via lowering the levels of several key pathogenic factors in the blood, including blood lipid, oxidation of low-density lipoprotein, and blood pressure (Gironi and Piemonte, 2011; Quideau et al., 2011; Camargo et al., 2019; Delgado et al., 2019; Michaličková et al., 2019).

## Biomass Polyphenols

**FIGURE 1:**
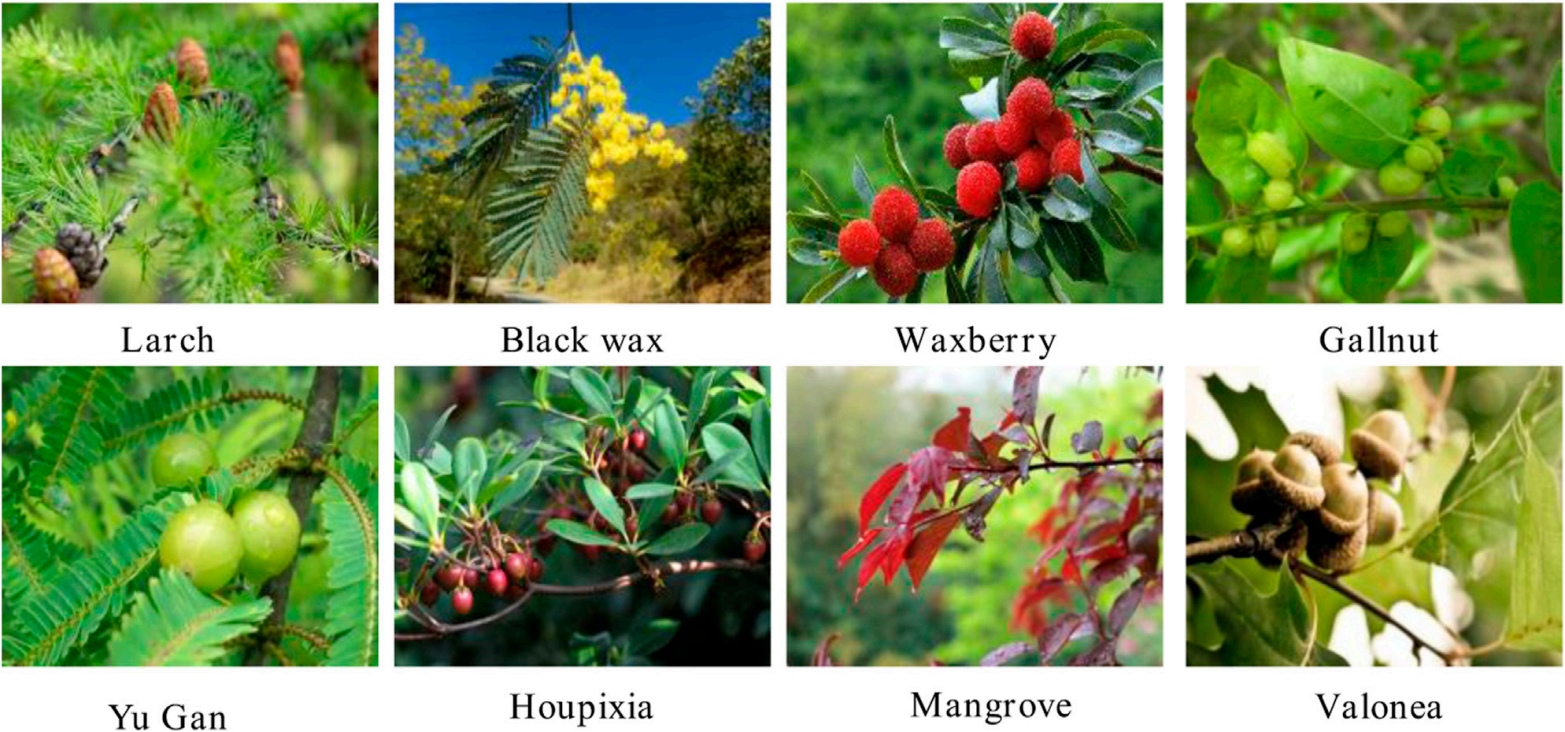
Different biomass that contain polyphenols.

Biomass polyphenols are a class of natural compounds widely distributed in biomass with an abundance second to lignin, cellulose, and hemicellulose. Polyphenols are predominantly accumulated in the leaves, vascular tissues, bark, immature fruits, seed coat, and disinfected tissues of biomass. China is rich in biomass polyphenol resources varieties (Figure 1), including Larch (100–150 mg/g, Yashunsky et al., 2014), Black wax (20–50 mg/g, Xu et al., 2016), Waxberry (20–50 mg/g, Chen et al., 2002), Yu Gan (200–300 mg/g, Yang and Liu, 2019), Houpixia (400–1,500 mg/g, Dai et al., 2006), Mangrove (500–600 mg/g, Dahibhate et al., 2020), Gallnut (300–500 mg/g, Ge et al., 2015).

**GRAPHICAL ABSTRACT:**
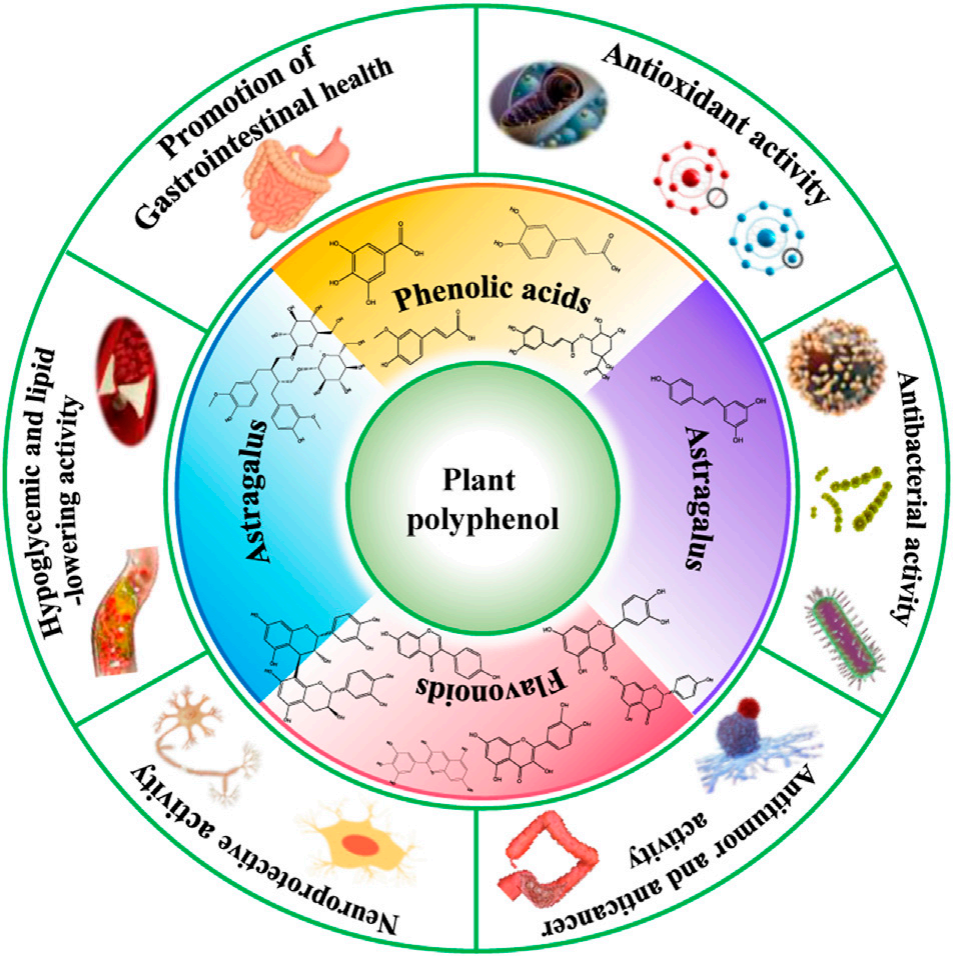


### Structure of Polyphenols

**FIGURE 2:**
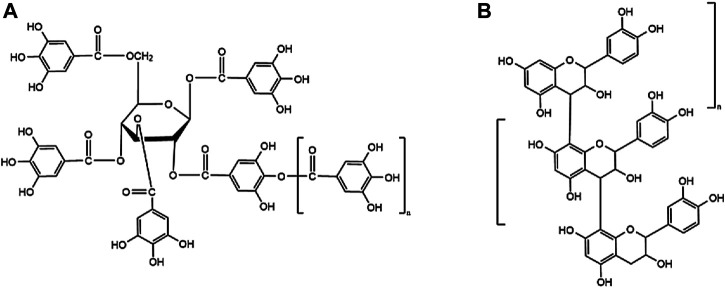
Structural representation of typical hydrolyzed tannins **(A)** and condensed tannins **(B)**.

Polyphenols share common structural features, their basic framework includes the polyhydroxy substitution of a benzene ring, as well as the absence of any nitrogen functional groups. Biomass polyphenols can be divided into the classes of 1) hydrolyzed tannins (gallate polyphenols) and 2) condensed tannins (polyflavanol polyphenols or proanthocyanidins) (Gironi and Piemonte, 2011). Hydrolyzed tannins are products of tannin hydrolysis, revolving around cleavage ester linkages. Condensed tannins are mainly composed of polyflavanol polyphenols or proanthocyanidins, which contain hydroxyl flavanol monomers connected by C-C bonds (Porter, 1992). Since hydrolyzed tannins and condensed tannins are completely distinct in the aspect of the unit skeleton, there are significant differences in their functional properties and applications (Figure 2). For example, hydrolyzed tannins are unstable and prone to be hydrolyzed under various conditions (acid, alkali, and under the presence of certain enzymes). Condensed tannins are not readily hydrolyzed, but can be further condensed into insoluble upon contact with a strong acid (Gessner and Steiner, 2005). When polyphenols interact with proteins, alkaloids, or polysaccharides, the polyphenol molecules initially approach the surface of protein molecules through hydrophobic bonds. The entry of polyphenols to the hydrophobic bag enables the following formation of multi-point hydrogen bonds. Due to a large number of coordination groups, most metal ions also tend to form precipitates if allowed to complex with polyphenols. Under alkaline conditions, polyphenols and metal ions readily form polycomplexes. In addition, the phenolic hydroxyl in the phenolic structure of biomass polyphenols (catechol or catechol) is easily oxidized to the quinone structure *via* consuming oxygen in the environment (Zhang et al., 2005).

### Classification of Polyphenols

More than 8,000 different kinds of polyphenols and their derivatives have been identified in the biomass kingdom (Boadas-Vaello et al., 2017). The name of polyphenols is assigned due to the presence of multiple phenolic groups in their chemical structures. In terms of structural differences, polyphenols can be further divided into four categories: phenolic acids, astragalus, lignans, and flavonoids (Table 1). A couple of studies have demonstrated the dominant biomass polyphenols that are found in common foods, including gallic catechins in green tea, resveratrol in grapes, capsaicin in chilis and peppers, curcumin in turmeric, genistein in soybean, and gingerol in ginger (Bhuyan, 2018). Investigation of the biological outcomes of these particular edible goods allows for a better understanding of the structural details related to the functionalities of polyphenols.

**TABLE 1:** Classification of polyphenols from different biomass.

Classification of polyphenols	Representative compounds	Structure	Biomass	References
Phenolic acids	Gallic acid	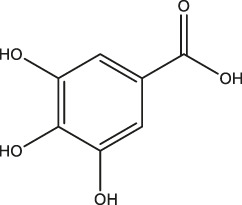	Gallnut, sumac, tea plant	Asnaashari et al., 2014; Wang et al., 2013
	Ferulic acid	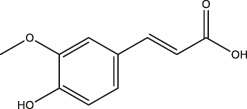	*Ferula*, ligustici, angelica	Zheng et al. (2021)
	Caffeic acid	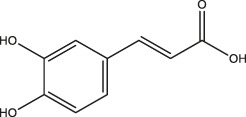	Coffee, Wine	
	Chlorogenic acid	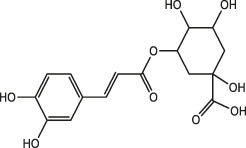	Honeysuckle, eucommia ulmoides leaves, hawthorn fruit	
*Astragalus*	Resveratrol	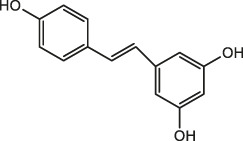	Peanut, mulberry, grape	Zheng et al., 2021; Hiradate et al., 2002
Lignans	Flaxseed lignans	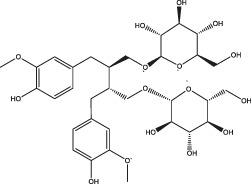	Flaxseed, sesame	Zheng et al. (2021)
Flavonoids	Luteolin, apigenin	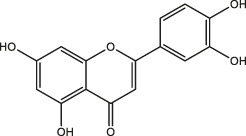	Parsley, dragonhead, Chili	Zheng et al. (2021)
	Quercetin, rutin	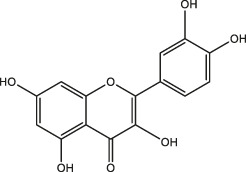	Apple, onions, Vegetables	
	Nobiletin, naringenin	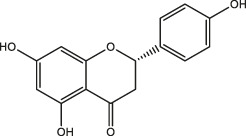	Citrus fruits	
	Daidzein, puerarin	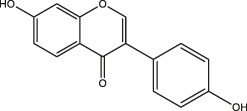	Legumes	
	Delphinidin, scabiolide	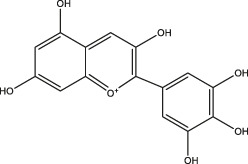	Fruits and vegetables with bright colors	
	Proanthocyanidin	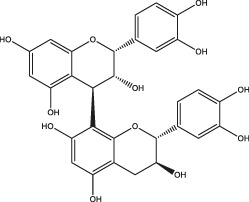	Blueberry, grape pip	

## Bioactivity of Polyphenols

Polyphenols are usually ingested as mixtures of different compounds that are immersed in a complex food substrate. The material then undergoes digestion, which exerts changes in structure and activity, before the mixture eventually reaches and acts upon target organs. After ingestion, absorption from the digestive tract usually requires intestinal enzymes, such as lactase rhizopericoside hydrolase and cytosolute β-glucosidase, to hydrolyze glycoside binders and produce the corresponding aglycones (Day et al., 2000; Gee et al., 2000). These aglycones can be further metabolized by second-stage enzymes to produce methylated, sulfurated, and gluconaldized compounds (Manach et al., 2004). Meanwhile, polyphenols that are not absorbed in the small intestine reach the colon, where they are converted into simpler metabolites by colonic microbiota and consequently being absorbed and get involved in further metabolic reactions (Liu et al., 2018).

Due to the diversity of biomass polyphenols, a variety of biological activities has been reported, including antioxidant (Hu et al., 2020; Ji et al., 2020), anti-inflammatory (Myint et al., 2021), bacteriostatic (Martin and Bolling, 2015; Gullon et al., 2016; Liu et al., 2019), anti-tumor (Sharma et al., 2017; Sajadimajd et al., 2020), regulation of intestinal flora (Cardona et al., 2013; Suzuki, 2013) and prevention of cardiovascular diseases (Kang, 2013; Tangney and Rasmussen, 2013; Kitai and Tang, 2017; Orr et al., 2020). Biomass polyphenols have also been widely used in the fields of the development of drugs and health products.

### Antioxidant Activity

**FIGURE 3:**
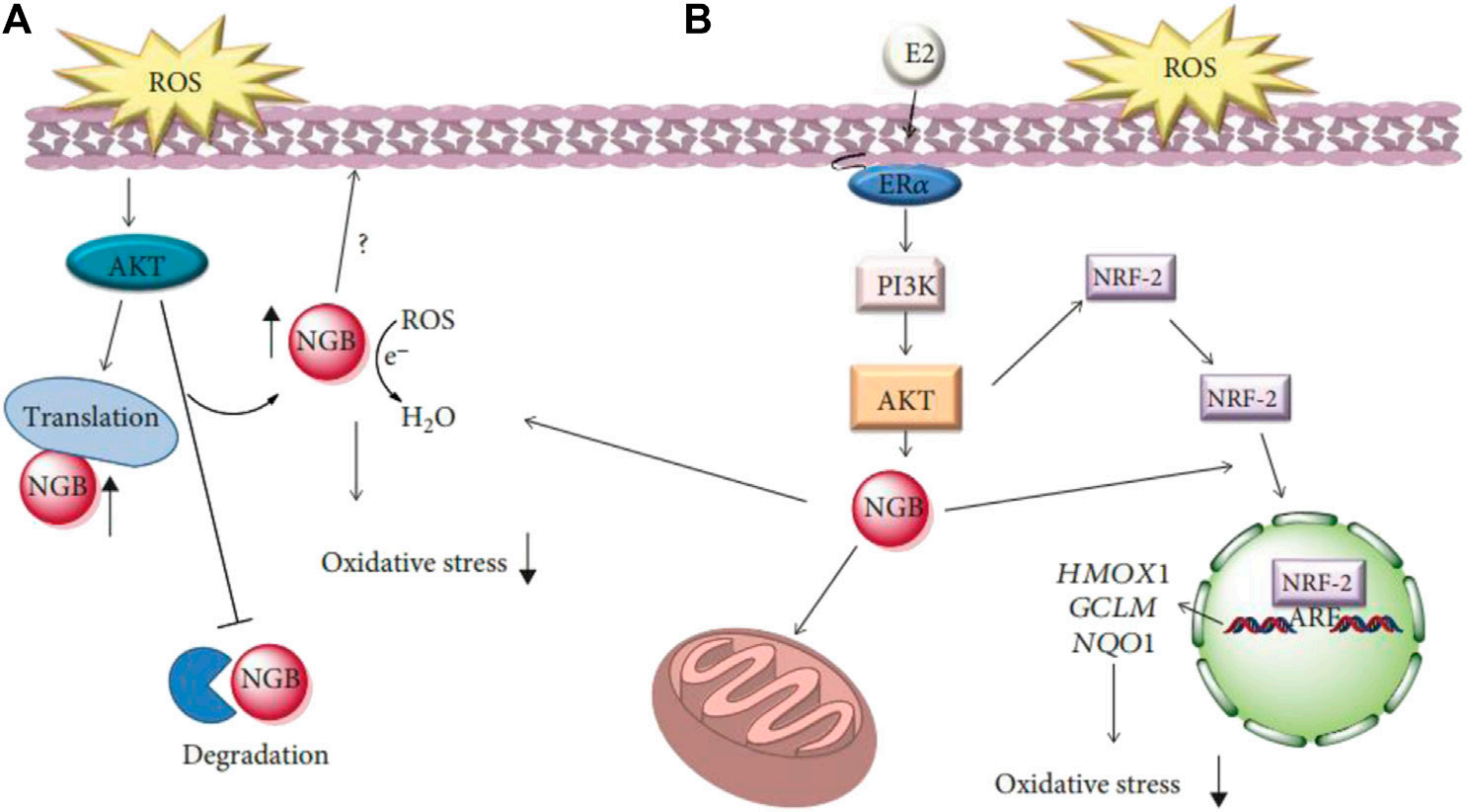
**(A)** Schematic model of ROS-activated signaling involved in the rapid modulation of neuroglobin (NGB) levels, its localization, and function on the redox balance outside mitochondria. **(B)** Schematic model of the impact of E2 intracellular-activated pathway on NGB expression, localization. The NRF-2 pathway describes how NGB affects the E2-dependent activation of the antioxidant NRF-2 system. E2: 17*β*-estradiol; ERα: estrogen receptor α; PI3K: phosphatidylinositol three kinase (Fiocchetti et al., 2019).

Redox is an essential class of metabolic reaction that occurs in living organisms. However, when the electron flow becomes decoupled, the generation of harmful free radicals results in detrimental outcomes (Fiedor and Burda, 2014; Zhao et al., 2021). Free radicals are atoms, molecules, or ions with unpaired electrons. They are highly unstable, will rapidly attack molecules in adjacent cells, and are prone to chemically react with other molecules (Yu et al., 2020). These reactions in turn contribute to various forms of impairments to cells. Most of the impairments can be repaired, but the entire reaction can be avoided if the free radical interacts with an antioxidant in cells. Antioxidants play a vital role in inhibiting molecular oxidation reactions to reduce the harmful accumulation of reactive oxygen species (Fiedor and Burda, 2014). Antioxidants also protect human;somatic cells from the deteriorating effects of free radicals and reactive oxygen species (ROS) by altering the expression of sensor proteins that are involved in oxidative stress (Figure 3) (Fiocchetti et al., 2019). The different kinds of chronic diseases and the process of lipid peroxidation are thus delayed. In recent years, there has been a great interest in unveiling natural plant-derived novel and safe dietary antioxidants.

Biomass polyphenols have strong activity due to their ability to delocalize uncoupled electrons, which can scavenge free radicals, chelate metal ions and inhibiting oxidase activity, and protect endogenous antioxidant enzymes in the body (Kim et al., 2014; Croft, 2016). Most natural antioxidants are phenolic compounds. The most important natural antioxidants are tocopherols, flavonoids, and phenolic acids. Among the phenolic hydroxyl groups, the phenolic hydroxyl group is the most easily oxidized, exhibiting the capacity to capture free radicals such as ROS and active nitrogen species (Geng et al., 2016; Zheng et al., 2021). This functionality enables polyphenols to scavenge free radicals and quench ROS, thus providing strong antioxidant capacity (Fraga, 2007; Dugasani et al., 2010; Losada and Dí az, 2017). These antioxidants, which are commonly used as food supplements, prevent the free radical chain reaction of oxidation and inhibit the initiation and propagation steps. All of these lead to the termination of the reaction and delay of the oxidation process. Antioxidants have the unique property of extending the shelf life of foods without any adverse effect on their sensory or nutritional qualities. Antioxidants used as food additives are non-toxic and effective at low concentrations. Other outstanding properties include high stability, robustness to the various stages of food processing, possess no smell, taste, or color, are easy to be mixed into foodstuffs, and have sufficient solubility.

Biomass polyphenols have been widely used in various fields due to their strong antioxidant activities. Hu et al. (2020) impregnated tea polyphenols (Gallic acid) into tea seed oil with ethanol and removed the ethanol by vacuum distillation to produce tea polyphenol colloids. It was found that no chemical changes occurred after the addition of tea polyphenols to tea seed oil. The antioxidant stability of colloidal tea polyphenols in tea seed oil was superior to that of synthetic antioxidants and tea polyphenol palmitate, and the optimal addition of tea polyphenols to tea seed oil ranged from 0.1–0.2 g/kg. Ji et al. (2020) found that the major phenolics in sea buckthorn were flavonoids, phenolic acids, and tannins, which showed antioxidant functions *via* regulating the activities of cellular enzymes. Myint et al. (2021) found that stevia leaves were demonstrated to possess the highest antioxidant capacity among plant foods due to the abundance of polyphenols (PPS). The stevia leaves PPS showed antioxidant activity similar to epigallocatechin gallate (EGCG), and their antioxidant activity, hydrophilic activity, and stability are stronger than ascorbic acid (VC), vitamin E, and chlorogenic acid. The antioxidant activity of stevia leaves PPS is stable under various physical conditions, except for in the presence of potassium sorbate or sucrose. In addition, the combination of PPS and VC improves their antioxidant stabilities. Taken together, PPS has the potential to be a natural, inexpensive, and abundant antioxidant for use in pharmaceuticals and cosmetics. In animal studies, Gerasopoulos et al. (2015) have included polyphenols extracted from olive oil processing wastewater to feed 20-day-old piglets for 30 days. The authors found that the polyphenol-rich diet significantly increased levels of total antioxidant capacity, catalase activity, and glutathione in the pig’s blood, as well as reducing oxidative stress. Liu et al. (2018) and Cimmino et al. (2018) showed that biomass polyphenols reduced the content of malondialdehyde in mutton, the fat oxidation was inhibited followed by the improvement of meat quality. More importantly, the biomass flavonoid polyphenol fisetin has been shown to relieve allodynia in a reserpine-induced rat model with fibromyalgia, hyperalgesia, and depression. Through evaluating multiple parameters, the researchers suggest that fisetin lowered biogenic amine (5-hydroxytryptamine, noradrenaline, and dopamine) levels, inhibited the oxidation of nitroso stress to downregulate ROS level, to exert its resistance to hurt feelings and antidepressant potential.

In conclusion, biomass polyphenols show prominent antioxidant performance and free radical scavenging capabilities, which is of great significance to broadening their fields of research and various applications.

### Antibacterial Activity

In recent years, consumers are increasingly intended to use natural extracts and other substances as potential antibiotics to inhibit the growth of pathogenic bacteria due to the concerns on the destruction of nutrition by sterilization technology and the abuse of synthetic antibiotics, as shown in Table 2 (Xu et al., 2019; Liu W. et al., 2020). Polyphenols are considered to be one of the intriguing natural extracts to hinder the growth and proliferation of bacteria *via* multiple modes of action, which include alteration of the bacterial membrane permeabilization, inhibition of the bacterial DNA gyrase, interference with the energy metabolism, and perturbation of the functions of bacterial porins (An et al., 2004; Gradisar et al., 2007; Wang et al., 2020; Yun et al., 2021). In addition, the presence of phenolic hydroxyl groups potentiates the antibacterial activities of polyphenols on damaging the structural integrity and functionality of bacterial membranes (Sousa et al., 2015).

**TABLE 2:** Antibacterial effect of different polyphenols.

Type of polyphenols	Biomass	Bacteria types	References
Polyphenol	Tea	*Proteus vulgaris, Staphylococcus aureus*	Pani et al. (2014)
	Apple	*Bacillus, Escherichia coli Pseudomonas, Bacillus subtilis*	Gullon et al. (2016)
	Pomegranate fruit slag	*Salmonella, Escherichia coli*	Salto and Lopez (2016)
Teucrium polium Flavonids	Teucrium polium	*Staphylococcus aureus*	Hafsa and Ibrahim (2017)
Oligomeric proanthocyanidins	Trester	*Streptococcus, Escherichia coli*	Hafsa and Ibrahim (2017)
Flavonoid	Olive	*Staphylococcus epidermidis*	Williams et al. (2017)
Polyphenol	Curry Leaves	*Staphylococcus aureus*	Hafsa and Ibrahim (2017)
Flavonoid	Hawthorn	*Staphylococcus aureus*	Yang and Zhang (2019)

Emerging evidence has demonstrated the beneficial effect of biomass polyphenols against bacteria. Pani et al. (2014) studied the toxicities of 29 polyphenols at different concentrations of the monophototoxin produced from *Fusarium* oxysporum in wheat. Most of the polyphenols exhibited an inhibitory rate of 70% against deoxynivalenol ranging from 1 to 1.5 mm. A serial of biomass polyphenols shows prominent inhibition on distinct strains of bacteria, fungi, and yeasts. Tea polyphenols are a kind of antimicrobial agent with a broad inhibitory spectrum on multiple pathogenic bacteria, such as *Proteus* common, *Staphylococcus* epidermidis, and *Staphylococcus aureus*. In addition, apple polyphenol extract elicits a suppression effect on the growth of *Bacillus aerobics*, *Escherichia coli*, *Pseudomonas*, and *Bacillus subtilis*. Pomegranate pulp is rich in eight different kinds of polyphenol compounds, all of which exert strong bacteriostatic abilities against *Salmonella* and *Escherichia coli* (Gullon et al., 2016). Flavonoids alone or in combination with known therapeutic agents effectively control *S. aureus* infection (Elmasri et al., 2015). More interestingly, Williams et al. (2017) found that oligoproanthocyanidins in grape dregs modulate intestinal microflora and alleviates intestinal *Ascaris suum* infection when grape dregs were added to the pig’s diet. When 10–40 g/kg grape seed powder was added to the chicken diet, it was found that the total number of *Streptococcus*, *Escherichia coli*, and microbial colonies in the chicken intestines decreased while the number of beneficial lactic acid bacteria increased in a dose-dependent manner (Hafsa and Ibrahim, 2017). Bearing this antibacterial activity in mind, researchers have endeavored to further investigate the mechanisms behind polyphenols’ bacteriostatic functionality.

**FIGURE 4:**
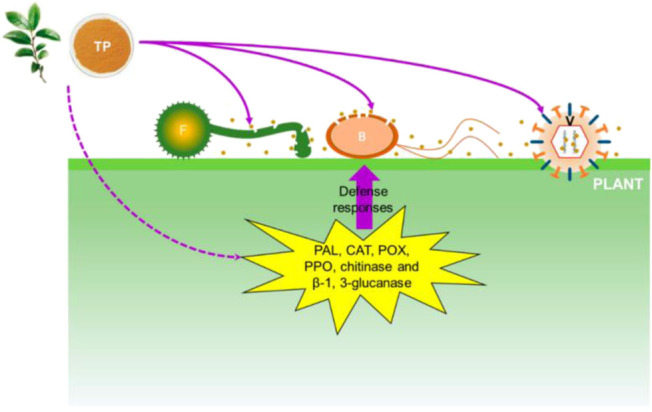
Antibacterial mechanism of polyphenols. Brown dots represent tea polyphenol. TP: tea polyphenol; F: fungi; B: bacteria; V: virus; PAL: phenylalanine ammonia-lyase; CAT: catalase; POX: peroxidase; PPO: polyphenoloxidase (Yang and Liu, 2019).

Yang and Zhang (2019) delineated the bacteriostatic mechanism of tea polyphenols as shown in Figure 4. Their results showed that the electrolyte leakage rate of bacteria was significantly enhanced after treatment with different concentrations of tea polyphenols, indicating the impairment of bacterial membrane permeability in the presence of polyphenols. The bacterial membrane is mainly composed of lipid bilayers containing hydrophilic and hydrophobic ends. The binding between phenolic hydroxyl groups and hydrophilic ends triggers agglomeration of membrane lipids, thus destroying the bacterial membrane. Intriguingly, when inoculated in plants and fruits, polyphenolic compounds induce the activities of a couple of antibacterial enzymes, including phenylalanine aminase, catalase, peroxidase, polyphenol oxidase, chitinase and β-1, 3-glucanase, thereby improving the antibacterial abilities of plants and fruits (Ramadas et al., 2020).

Currently, the studies on the antibacterial properties of biomass polyphenols continue to be carried out in breadth and depth. However, the structure-function relationship of polyphenols and the combinational applications of polyphenols with more prominent antibacterial effects require further investigation.

### Antitumor and Anticancer Activity

**FIGURE 5:**
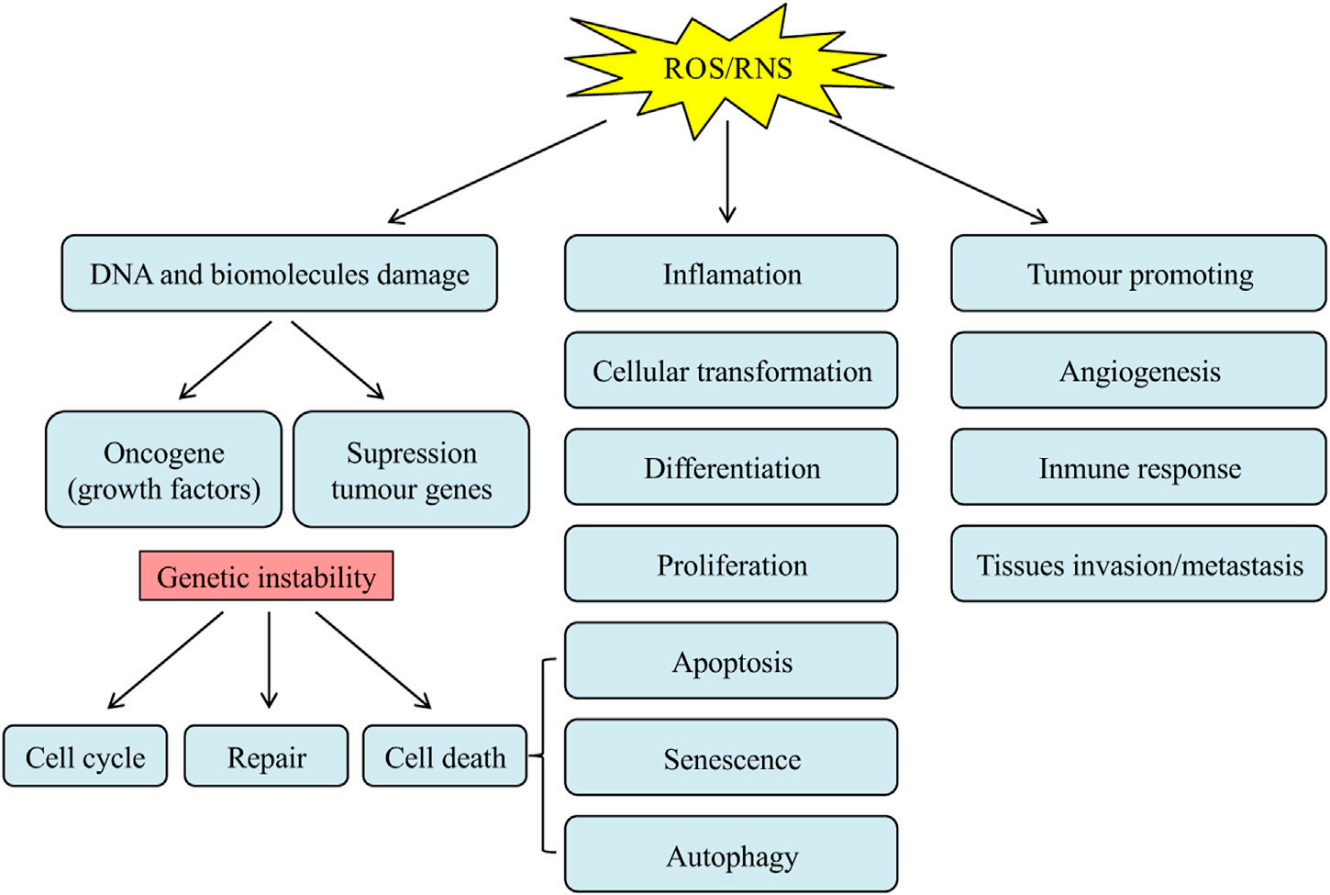
The role of ROS/RNS in carcinogenesis (Ríos-Arrabal et al., 2013).

Reactive oxygen free radicals are the metabolites of the Redox reaction in biological organisms. Under normal physiological conditions, the generation and scavenging of free radicals are finely balanced in a dynamic equilibrium. However, when the imbalance occurs, excessive free radicals will deteriorate the organisms, leading to aging and the increased incidence of a range of disorders (Zhang et al., 2020; Wang et al., 2021). The accumulation of free radicals generates direct damage on the genetic materials and other biological macromolecules, including the aberrant gene transcriptional activation, changes in the structural and functional identities of proteins, breakage, and polymerization of peptide bonds, and lipid peroxidation, leading to the occurrence of tumors and cancers (Figure 5) (Ríos-Arrabal et al., 2013). Polyphenols have attracted broad attention in cancer therapeutics due to their chemopreventive roles as both blocking and suppressing agents (Sharma et al., 2017; Sajadimajd et al., 2020). In terms of their blocking functions, polyphenols can avoid the activation of carcinogens, prevent the reactive carcinogens from interacting with critical DNA sites, and facilitate the metabolic clearance of carcinogens. Moreover, polyphenols are capable of suppressing oncogenesis and cancer progression, to elicit their chemopreventive functions on multiple stages of carcinogenesis (Zhou et al., 2016).

**FIGURE 6:**
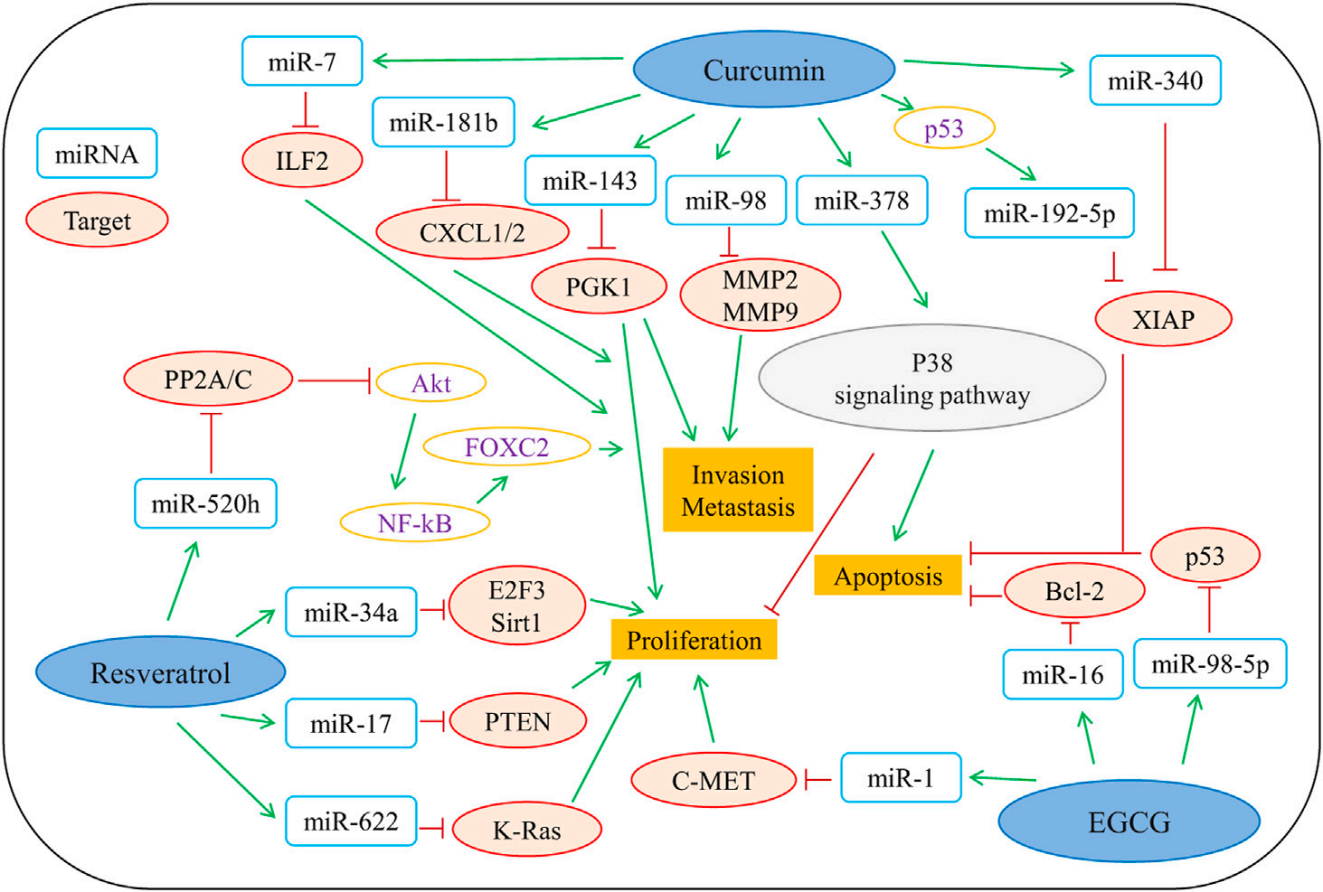
Representative polyphenols that are involved in regulating the antitumor mechanisms of microRNAs. MiR, microRNA; ILF2, Interleukin enhancer binding factor 2; CXCL1/2, chemokine (C-X-C motif) ligands 1/2; PGK1, phosphoglycerate kinase 1; MMP2/9, matrix metalloproteinase 2/9; XIAP, X-linked inhibitor of apoptosis; PP2A/C, protein phosphatase 2A/C; E2F3, E2F transcription factor 3; Sirt1, Sirtuin type 1; PTEN, phosphatase and tensin homolog; K-Ras, kirsten rat sarcoma; C-MET, cellular-mesenchymal epithelial transition factor; Bcl-2, B-cell lymphoma-2; p53, protein 53; P38 signaling pathway, protein 38 signaling pathway. reproduced with copyright permission from Elsevier (Yi et al., 2019).

Vegetables and fruits contain a wide variety of polyphenols, and studies have reported that regular consumption of fruits, vegetables, and nuts reduces the risk of various types of cancer, especially with a significant impact on gastric, esophageal, lung, oral, pharyngeal, pancreatic and colon cancers (Yi et al., 2019). Epidemiological and experimental studies have shown that consumption of food and beverages rich in polyphenols (such as catechins, flavonoids, and anthocyanins) is closely associated with a lower incidence of cancer (Naasani et al., 2003). Animal experiments have also demonstrated that food polyphenols effectively suppress chemical-induced tumors and inhibit tumor developments at multiple stages (Sharma et al., 2017; Sajadimajd et al., 2020). An increasing number of studies highlight the role of biomass polyphenols as potential anticancer cell mutagens. Han et al. (2019) showed that cranberry-extracted polyphenols are bioactive anticancer components, and they have dramatic capacities towards inhibiting the viability and colony formation of human colon cancer cells HCT116. Mechanistically, treatment of polyphenols caused the cell cycle arrest at G0/G1 phase and subsequently led to the induction of cell apoptosis. There is ample evidence showing that polyphenols target a variety of molecules that are involved in multiple cellular signaling pathways. Emerging evidence has shown that non-coding RNAs function as oncogenes or tumor suppressors in the regulation of tumorigenesis and tumor progression (Yi et al., 2019). The antitumor mechanisms of polyphenols are multi-targeted and include the activation of different pathways to induce apoptosis in cancer cells. Moreover, three predominant epigenetic changes (alterations in chromatin structure, DNA methylation, and regulation by microRNAs) are also involved in tumor cells treated with biomass polyphenols. As shown in Figure 6 (Yi et al., 2019), EGCG, curcumin, and resveratrol regulate multiple classes of miRNAs to elicit their antitumor potentials.

Although multiple targets have been identified in terms of the antitumor/anticancer activities of the biomass polyphenols, the detailed mechanisms of how polyphenols are capable of controlling the expression of these genes/miRNAs remain elusive. It would therefore be interesting to select single or high purity polyphenols from natural product resources with strong antitumor/anticancer activities to further investigate their relationships with antitumor factors.

### Neuroprotective Activity

The incidence of neurodegenerative disorders, such as Alzheimer’s disease and Parkinson’s disease, gradually increases with age (Remington et al., 2010; Figueira et al., 2017; Li, 2018). These types of disorders share common pathological hallmarks, including oxidative stress, neuroinflammation, protein aggregation, and mitochondrial dysfunction (Gu et al., 2021). Given their roles in mediating essential biological processes, including signal transduction, cell proliferation and apoptosis, and cell differentiation, polyphenols have been long taken as potential neuroprotective agents. More importantly, the neuroprotective function of polyphenols has been suggested to be associated with their antioxidant activities, especially towards scavenging ROS and nitric oxide (Zhen and Liu, 2018).

**FIGURE 7:**
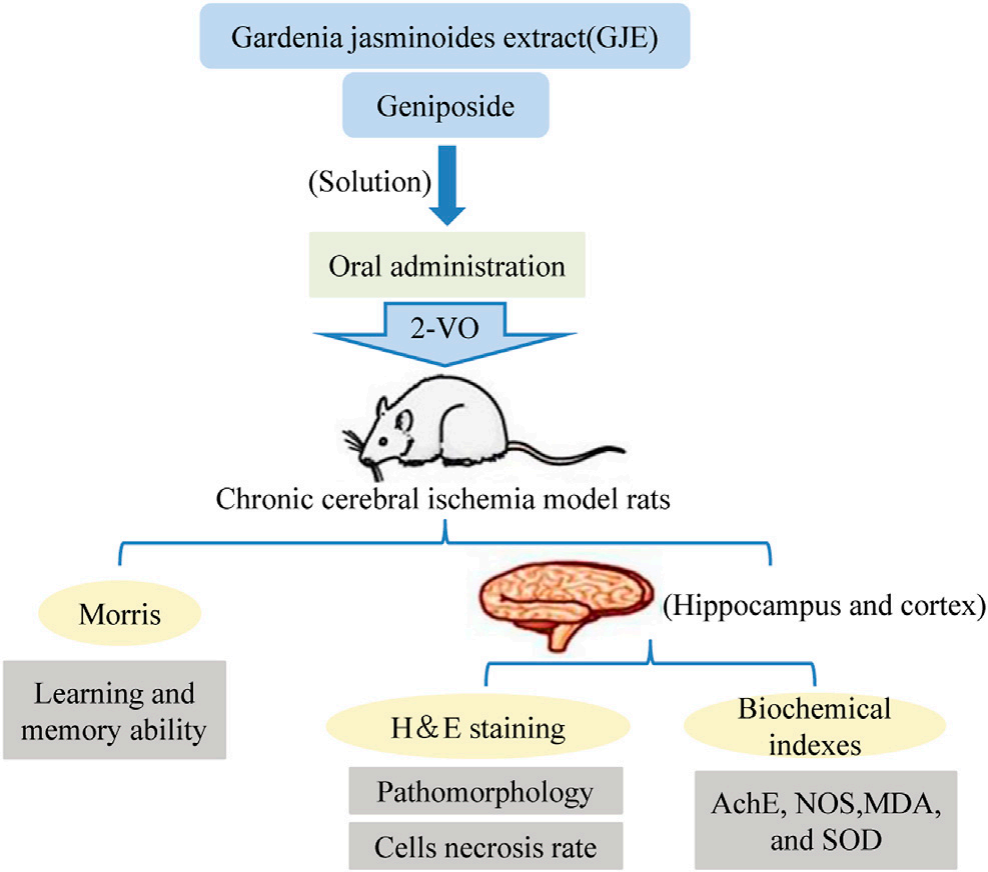
The neuroprotective roles of *Gardenia jasminoides* extract (GJE) and Geniposide on a rat model with chronic cerebral ischemia (Zhang et al., 2016).

A growing number of studies have provided experimental evidence that the consumption of polyphenol-rich berry fruits is beneficial to the nervous system and shows the potential to mitigate age-dependent neurodegeneration *via* alleviating cognitive and motor dysfunctions (Figueira et al., 2017; Tavares et al., 2013). Moreover, the neuroprotective function has also been demonstrated on *Gardenia jasminoides* extract (GJE). The medium dose of GJE treatment showed the most effective inhibition of neuronal necrosis in different brain regions of the rat model of chronic cerebral ischemia (Figure 7) (Zhang et al., 2016). Green tea polyphenols have been demonstrated to play a neuroprotective role due to their antioxidant and anti-inflammatory properties (Sutherland et al., 2006; Song et al., 2019). Zhang et al. (2010) showed that a 30-days treatment with green tea polyphenols (200 mg/kg, twice a day) prominently restored blood-brain barrier permeability, rescued cerebral infarction and improved neurological functions in rats underwent cerebral ischemia. Moreover, the induction of caveolin-1 mRNA and hyperphosphorylation of extracellular signal-regulated kinase 1/2, markers of cerebral ischemia, were also found ameliorated in cerebral ischemic tissue. Liu et al. (2019) isolated four catechins, including two new catechin derivatives, from Anhua dark tea. The study showed that the compounds exhibited optimal neuroprotective effects by inhibiting N-methyl-p-aspartate (NMDA) receptors. It protected SH-SY5Y cells from NMDA-induced injury and apoptosis by regulating NR2B expression and activating PI3K/Akt signaling pathway. These compounds are expected to be effective therapeutic agents for the prevention of excitatory brain injury. Taken together, these findings emphasize that the antioxidant, anti-apoptotic, and reduction of brain edema activities of tea polyphenols are prerequisites for their neuroprotective functions.

### Hypoglycemic and Lipid-Lowering Activity

High blood lipid content is one of the essential risk factors of fatty liver, cerebral infarction, coronary heart disease, and the formation of vascular sclerosis. Excessive accumulation of blood glucose in diabetic patients easily leads to acute severe metabolic disorders, for example, life-threatening hyperosmolar hyperglycemia syndrome. In the meantime, diabetic patients also suffer from infectious diseases, which lead to chronic complications including microangiopathy, diabetic nephropathy, and diabetic retinopathy. The nervous system complications may also be accompanied, such as peripheral neuropathy, autonomic neuropathy, and diabetic feet. All of these symptoms and complications severely ruin the quality of life of diabetic patients (Monika L. et al., 2019). Diabetic patients with combined neuropathy also develop pancreatic sclerosis and atrophy (He et al., 2020). Medicinal biomass has been applied to control diabetes and hyperlipidemia in different countries (Sugiyama et al., 2007; Yang et al., 2010; Saeed et al., 2012), and has become the major source of safe and effective hypoglycemic and hyperlipidemic drugs. Importantly, the hypoglycemic activity has been assigned to biomass polyphenols due to their capabilities in exerting antioxidant functions, promoting the synthesis and secretion of insulin, perturbing the activities of intestinal digestive enzymes, and inhibiting the glucose transport (Mahmood et al., 2013).

**FIGURE 8:**
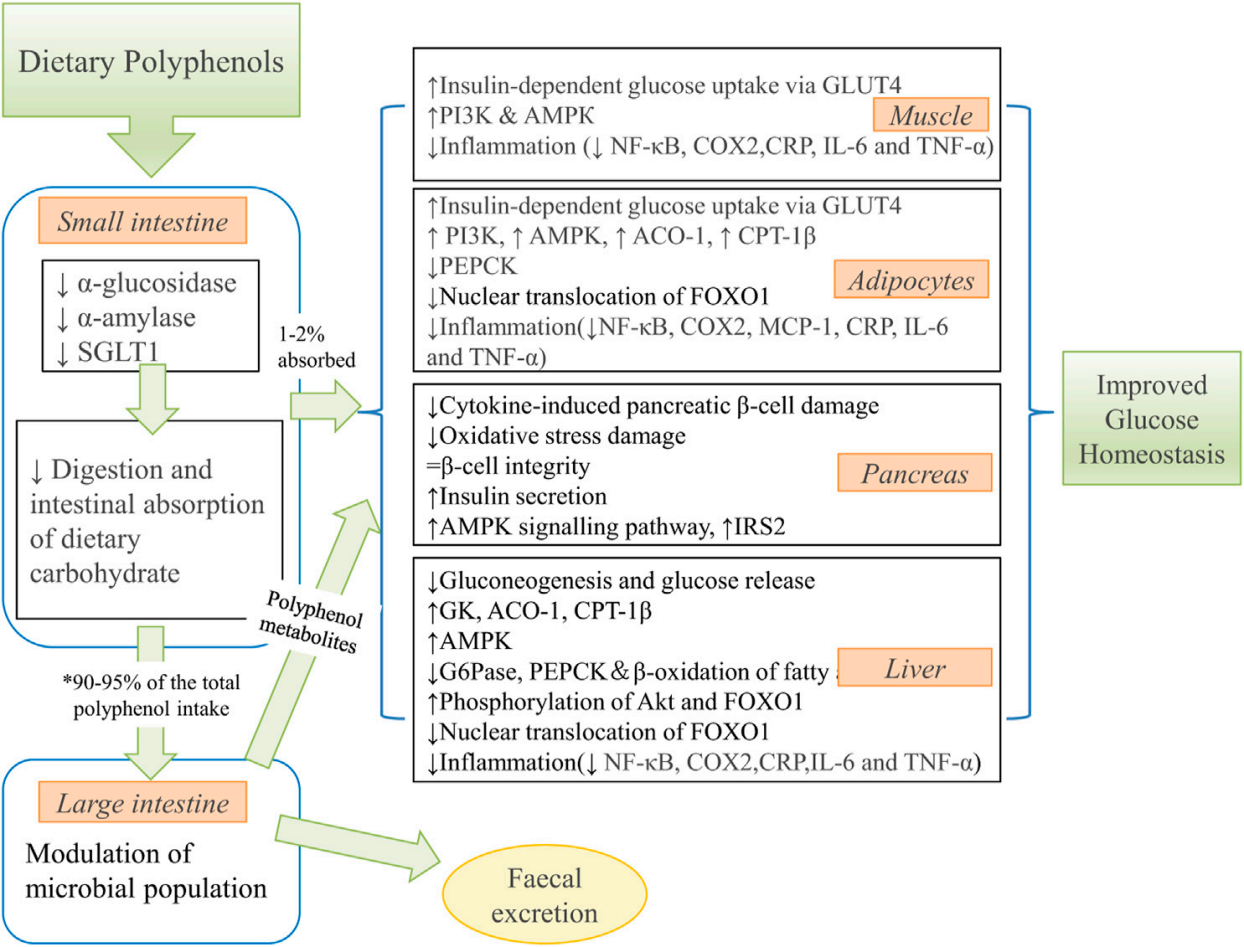
The summary of potential mechanisms linking dietary polyphenol metabolites to improved glucose homeostasis. ↑, increase; ↓, decrease. * 90–95% of the ingested polyphenols reach the colon. SGLT1, sodium-dependent glucose transporter; GLUT4, glucose transporter four; PI3K, phosphoinositide 3-kinase; AMPK, 5′ adenosine monophosphate-activated protein kinase; NF-κB, nuclear factor kappaB; COX2, cyclooxygenase-2 protein; CRP, C-reactive protein; IL-6, interleukin 6; TNFα, tumor necrosis factor α; ACO-1, acyl CoA oxidase-1; CPT-1β, carnitine palmitoyl transferase-1β; PEPCK, phosphoenolpyruvate carboxykinase; FOXO1, forkhead box protein O1; MCP-1, monocyte chemoattractant protein-1; IRS2, insulin receptor substrate two; GK, glucokinase; G6Pase, glucose-6-phosphatase. (reproduced with copyright permission from MPDI (Kim et al., 2016)).

Chakraborty et al. (2012) emphasized that the role of 6-gingerol in controlling insulin responsiveness *via* regulating insulin secretion of mouse pancreas is essential for protecting the hyperglycemia and oxidative stress caused by arsenic. When the mice were fed with 6-gingerol for 12 days, Singh et al. (2009) reported a significant reduction of fasting blood glucose, accompanied by increased glucose tolerance and downregulation of plasma triglyceride (TG), total cholesterol (TC), insulin, low-density lipoprotein cholesterol (LDL-C) and free fatty acid (FFA) levels. These findings support the anti-hyperglycemia and cholesterol-lowering activities of 6-gingerol. The other kinds of biomass polyphenols function to increase insulin sensitivity and improve insulin resistance. Manzano et al. (Manzano et al., 2016) showed that apple polyphenols (APE, mainly quercetin and rutin) have therapeutic potential in the rat model of insulin resistance. Nutritional intervention with APE resulted in increased insulin sensitivity and a 45% increase in glucose infusion rate (GIR). Furthermore, *in vitro* results showed a synergistic effect between APE and insulin to increase glucose uptake through GLUT4 translocation in muscle cells. This translocation is mediated by the phosphatidylinositol 3-kinase (PI3K) and peroxisome proliferator-activated receptor-γ (PPARγ) signaling pathways. Xiong et al. (2020) also demonstrated that catechins, procyanidin Al and procyanidin A2 extracted from lychee seed LSF could activate the insulin signaling pathway and inhibit GSK-3β activity *via* the IRS-1/PI3K/Akt pathway, which in turn inhibited Tau hyperphosphorylation and ultimately improved cognitive function in AD rats. Kim et al. (2016) provided a summary of potential mechanisms by which dietary polyphenol metabolites improve glucose homeostasis (Figure 8).

Vroegrijk et al. (2011) found that male C57BL/J6 rats fed with a high-fat diet containing 1% pomegranate seed oil for 12 weeks showed reduced fat content and body weight compared with those fed with a full-fat diet. Additionally, Xu et al. (2009) studied the effect of pomegranate flower extract on hepatic fat accumulation in Zucker diabetic obese rats with severe fatty liver disease and highlighted the hypolipidemic effect of the isolated polyphenols. Yu et al. (2014) found that pomegranate leaves (PGL) have a similar modulating effect on lipid metabolism. Pomegranate leaves and their major active components (ellagic acid, gallic acid, pyrogallic gallic acid, and tannic acid) showed the effect of inhibiting pancreatic lipase activity *in vitro*. High doses of PGL inhibited intestinal lipase activity while promoting the expression of tight junction proteins, thereby inhibiting lipid absorption and reducing blood serum total cholesterol (TC) and triglyceride (TG) levels to prevent intestinal mucosal damage due to lipid overload.

Green tea polyphenols, grape polyphenols, citrus juice polyphenols, and sand buckthorn leaf polyphenols play similar roles in lowering blood sugar *via* multiple modes of action. In addition, several common fruit and vegetable polyphenols, such as pomegranate polyphenols, tea polyphenols, hawthorn polyphenols, and apple polyphenols, exert similar effects on downregulating TG, TC, and LDL-C levels while upregulating the level of high-density lipoprotein cholesterol. Currently, the study on the mechanisms of glucose- and lipid-lowering capabilities of biomass polyphenols has attracted much more attention but still requires further investigations.

### Promotion of Gastrointestinal Health

Intestinal barriers refer to the intact structure and function of the intestine to prevent harmful substances such as bacteria and toxins from passing through the intestinal mucosa and entering other tissues, organs, and blood circulation in the human body. The normal intestinal mucosal barriers are composed of a mechanical barrier, a chemical barrier, an immune barrier, and a biological barrier, and the integrity of each intestinal barrier is indispensable to human health. The intestinal barriers maintain the normal intestinal permeability and regulate the transportation and absorption of nutrients (such as sugar, vitamins, amino acids, fatty acids, and other lipids) and other food-related compounds (such as polyphenols). In addition, intestinal barriers regulate the composition of bacteria from the lumen to the blood flow of transfer (Tangney and Rasmussen, 2013; Hidalgo-Liberona et al., 2020). The intestinal permeability is under control of a complex system of junctions known as tight junctions (TJ), gap junctions, and adhesion junctions. The system has consisted of numerous TJ proteins and junction adhesion molecules that control the flow among adjacent intestinal cells. It has been reported previously that polyphenols can mitigate leaky bowel disease by directly adjusting TJ function, enhancing the synthesis and redistribution of TJ proteins (such as occludin, claudins, and occludula), and suppressing the activities of different kinases involved in controlling TJ expression (Hidalgo-Liberona et al., 2020).

**FIGURE 9:**
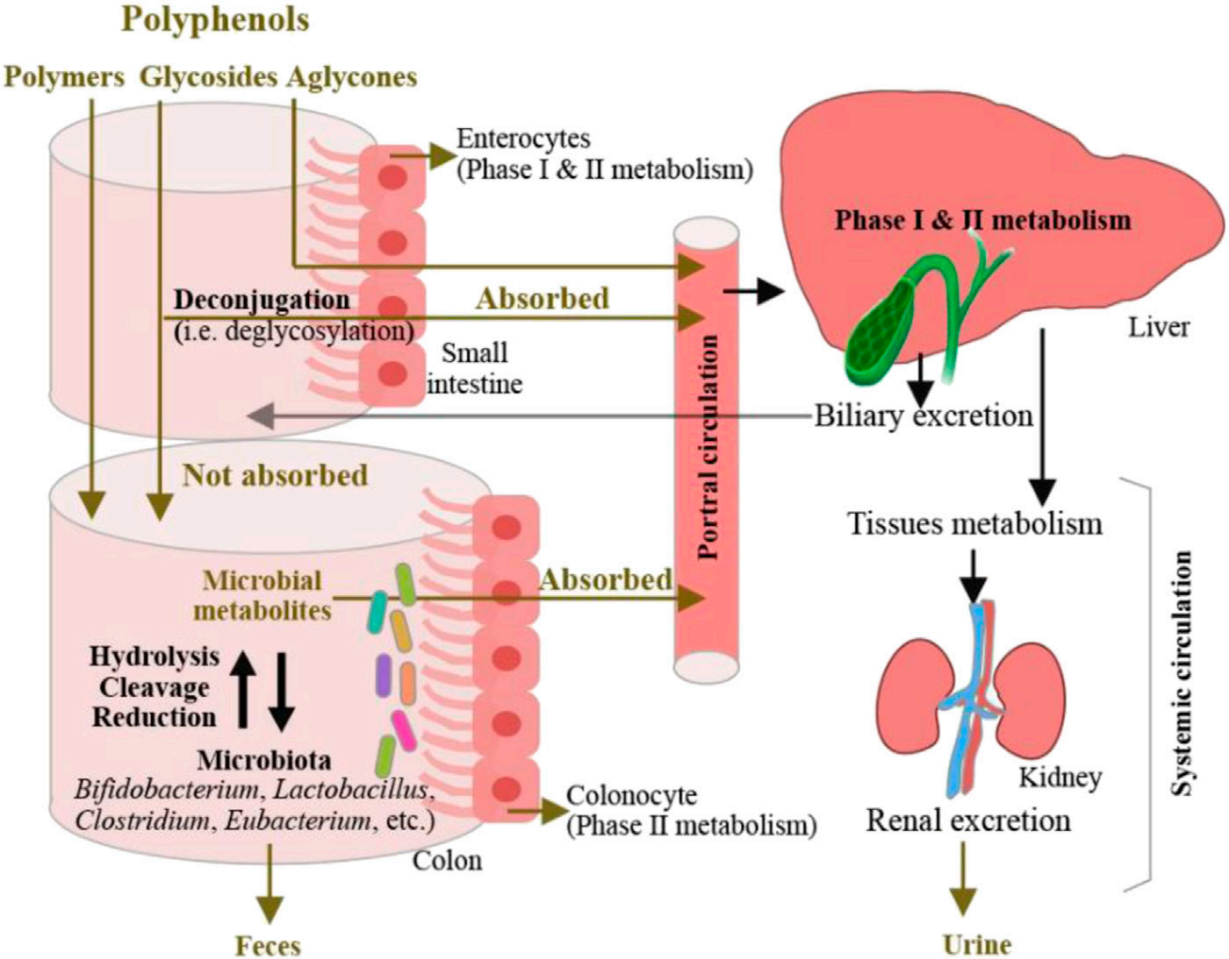
Absorption of dietary polyphenols and their microbial transformation in the intestine. [reproduced with copyright permission from ACS Publications (Liu J. et al., 2020)]

Gastrointestinal dysfunction is one of the major factors that contribute to type II diabetes, cardiovascular disease, insomnia, obesity, and other disorders (Kang, 2013; Kitai and Tang, 2017; Orr et al., 2020). Therefore, improvement of gastrointestinal function requires much more investigation. Previous studies (Pandey and Rizvi, 2009) have reported that polyphenols show antioxidant, anti-inflammatory, anti-fat, anti-diabetes, cardioprotective, neuroprotective, and anticarcinogenic effects *via* collaborating with the intestinal microbiota. Biomass polyphenols influence the activities of intestinal microflora, repair gastrointestinal mucosal damage, optimize the intestinal structure, and interact with other macromolecules to affect gastrointestinal function. Chen et al. (2018) showed that the addition of chlorogenic acid to weaning piglets led to an increase of immune globulin level, the expression of antiapoptotic protein B-cell lymphoma-2 was simultaneously upregulated in the duodenum and jejunum. This indicates that the intestinal beneficial effect of chlorogenic acid depends on the enhancement of immune function and suppression of excessive intestinal epithelial cell apoptosis. Liao et al. (2016) showed that upon treatment of tea polyphenols, the reduction of atherosclerosis plaque in mice negatively correlated with the increased number of bifidobacteria in their intestine, suggesting that tea polyphenols promote the proliferation of bifidobacteria and prevent lipid metabolism, thereby suppressing atherosclerosis. Biomass polyphenols were found to accelerate beneficial bacteria proliferation to improve the function of the intestines and stomach and repair the damaged intestinal cells. Zhao et al. (2018) showed that when treated with bitter butyl tea polyphenols, the reduction of gastric acid secretion and increase of gastric juice pH was detected in mice with gastric mucosa damage, indicating that bitter butyl tea polyphenols supplement was an effective approach to combat against gastric mucosa damage. With the increasing number of studies on the relationship between biomass polyphenols and gastrointestinal function, the development and utilization of biomass polyphenols as functional factors for the improvement of gastrointestinal function is expected to be broadened. Liu et al. (2020a) depicted the metabolic mechanisms of dietary polyphenols in the intestine (Figure 9). In the body, a small percentage of dietary polyphenols is first absorbed in the small intestine. They are then deconjugated, circulated, and distributed among organs or excreted in the urine. The remaining unabsorbed polyphenols reach the colon where they are catabolized by bacteria to produce metabolites either absorbed or excreted in feces. After intestinal and hepatic Phase I and II metabolism, the microbial-derived polyphenolic metabolites enter the systemic circulation. The metabolites in the liver could be excreted *via* the biliary duct and re-absorbed throughout the enterohepatic recirculation. In animal studies, the addition of polyphenols to diets reduced high-fat diet-induced obesity and modulated the gut microbiota by increasing the growth of short-chain fatty acid-producing bacteria and decreasing the growth of lipopolysaccharide-producing bacteria. More clinical trials are required to investigate the application of dietary polyphenols as nutritional or functional foods in the prevention and treatment of obesity in humans, and studies that aim at elucidating the mode of action of specific bacteria strains in mediating dietary polyphenols would be necessary.

## Conclusion and Perspectives

Although polyphenols have been considered as chemical impurities, recent studies and findings underlined its biological activities in terms of exerting antioxidant, antibacterial, antitumor, neuroprotection, regulation of blood lipid, and promotion of gastrointestinal health functions. This thus attracts more attention from researchers worldwide to further investigate the pharmacological applications of biomass polyphenols and use them as one of the major components in natural products-derived drugs. More interestingly, given that biomass polyphenols are enriched in daily food, this further highlights the essential contribution of polyphenols to human life and makes biomass polyphenols one of the research hotspots. A range of studies has demonstrated efficient extraction of polyphenols from tea, grape, pomegranate, rapeseed, and other raw materials, which are coincidentally used in medical treatments and as functional food supplements. Many diseases are associated with antioxidants, but given the purity of the extract and the complexity of the structure, polyphenols are currently only used as supplements for the treatment of diseases, and research into their use as medicines for the treatment of diseases still requires innovative extraction techniques and in-depth research into anti-disease mechanisms, to explore their therapeutic potential. Hence, the mechanisms of polyphenols’ pharmacological actions still require further investigation. It is hoped that with the increasing attention from researchers on natural drugs and the progress of scientific technology, more methods of rapid separation and preparation of polyphenols can be developed, and the underlying pharmacological mechanisms of polyphenols will be further elucidated to provide the material basis for further pharmacological examination and clinical investigation.
